# Left and right displaced abomasum and abomasal volvulus: comparison of clinical, laboratory and ultrasonographic findings in 1982 dairy cows

**DOI:** 10.1186/s13028-022-00656-9

**Published:** 2022-12-20

**Authors:** Ueli Braun, Karl Nuss, Sarah Reif, Monika Hilbe, Christian Gerspach

**Affiliations:** 1 Department of Farm Animals, Vetsuisse Faculty, University of Zurich, Zurich, Switzerland; 2 Institute of Veterinary Pathology, Vetsuisse Faculty, University of Zurich, Zurich, Switzerland

**Keywords:** Abomasal displacement, Abomasal volvulus, Cattle, Diagnosis, Findings

## Abstract

**Background:**

Although left and right displacement of the abomasum and abomasal volvulus are well-known disorders of cattle, a comparative evaluation of the clinical, laboratory and ultrasonographic findings of these types of abomasal displacements has not been undertaken. Therefore, the objective of this study was to compare these conditions in a large population of cows. The medical records of 1982 dairy cows with left displaced abomasum (LDA, n = 1341), right displaced absomasum (RDA, n = 338) and abomasal volvulus (AV, n = 303) were searched for the results of clinical, laboratory and ultrasonographic evaluations.

**Results:**

The main clinical findings were an abnormal demeanour in 48.2% of the cows, reduced rumen motility in 89.7% and decreased intestinal motility in 61.1%. Ballottement and simultaneous auscultation and percussion and simultaneous auscultation were positive on the left side in 96.9% of the cows with LDA and on the right in 98.5% of the cows with RDA and in 99.3% of the cows with AV. Ultrasonography was useful for diagnosing LDA in 97.9% of the cows and RDA/AV in 90.2% of the cows. The laboratory findings characteristic of abomasal reflux syndrome varied in severity; 83% of the cows had hypokalaemia, 67% had increased rumen chloride concentration, 67% had an increased base excess and 50% had haemoconcentration. Based on the clinical signs, a definitive diagnosis was made in 75.0% of the cows with LDA and in 22.5% of the cows with RDA/AV. Ultrasonography was required for a definitive diagnosis in another 22.0% of the cows with LDA and in 53.0% of the cows with RDA/AV. Laparotomy or postmortem examination was required for reliable differentiation of RDA and AV.

**Conclusions:**

LDA, RDA and AV differ with respect to the severity and the frequency of several abnormal clinical, laboratory and ultrasonographic findings as well as the methods required for a diagnosis.

## Background

The clinical and laboratory findings in cows with left (LDA) and right displaced abomasum (RDA) and abomasal volvulus (AV) have been described in textbooks [1–4], reviews [5–9] and scientific publications [10–19]. Likewise, the ultrasonographic findings in cows with LDA [20–22] and RDA/AV (term used collectively for cows with either RDA or AV) [23] have been reported. Several authors have examined the prognostic utility of clinical and laboratory variables in the diagnosis of displaced abomasum and abomasal volvulus [11, 12, 15, 24–27]. In cows with acute LDA, the demeanour is often only mildly abnormal and the rectal temperature and the heart and respiratory rates are within or near the reference intervals [4]. However, as the duration of LDA increases, cows become anorexic and milk production decreases sharply [2]. Rumen motility is often reduced or absent and the rumen sounds become muffled because of interposition of the abomasum between the rumen and the left abdominal wall [2, 4, 9]. Spontaneous abomasal sounds may be audible over the cranial abdominal wall [1]. Auscultation, while air is blown into the rumen through a stomach tube, may aid in the differentiation of the rumen and the displaced abomasum [1, 4]. Ballottement and simultaneous auscultation (BSA) and percussion and simultaneous auscultation (PSA) of the left abdominal wall are positive, i. e., they generate fluid-splashing sounds caused by the movement of fluid (BSA) and ringing sounds (pings, steel band effects) caused by gas in the abomasum (PSA) [1, 2, 28].

Transrectal abdominal exploration of a cow with LDA may show medial displacement of the rumen [2, 4, 9] or a taut fold of the greater omentum enclosing the caudal recess of the omental bursa [1]. The displaced abomasum may be palpated transrectally in no more than about 2% of all cases [1]. Faecal output is usually reduced [29] but faecal consistency is normal in most cases [14]. Both the demeanour and initial clinical signs of cows with acute RDA are similar to those of cows with LDA [29]. Ballottement and simultaneous auscultation and PSA are positive on the right side of the cow in the 9th to 12th intercostal spaces [5], and the right side of the abdomen may be dilated [2]. Rumen motility is significantly reduced [7], and the displaced abomasum may [12] or may not be palpable transrectally [7]. The demeanour of cows with AV is more severely affected than cows with LDA or RDA and their condition becomes worse as the degree of the volvulus increases. Signs of colic are common in the initial stages [1] followed by lethargy [5]. Tachycardia with heart rates exceeding 100 beats per minute (bpm) is common, and severe dehydration causes enophthalmos [5]. The body temperature decreases with an increase in the severity of the illness [11, 14], and rumen motility is severely reduced or absent [2]. BSA and PSA are positive on the right side. The faeces appear dark brown and greasy, have a pasty consistency and are reduced in amount [5]; often the rectum is empty. Transrectal palpation of the displaced abomasum in the upper right quadrant of the abdominal cavity was reported to be possible in 57.5% of 55 cows [12] and in 76.3% of 80 cows [25]. Clinical differentiation of RDA and AV is not always possible [5]. Abomasal reflux syndrome is the most important factor in the pathogenesis of disorders associated with abomasal displacement [30]. Abomasal displacement is associated with gastrointestinal obstruction, which causes reflux of abomasal contents, including hydrogen and chloride ions, into the rumen [30]. The result is an increase in rumen chloride concentration, with concentrations of up to 100 mmol/L [2], and hypochloraemia because small intestinal resorption of chloride ions is impaired. This in turn leads to a decrease in the secretion of bicarbonate into the small intestines and an increase in the bicarbonate concentration in the blood resulting in metabolic alkalosis. Initial compensatory mechanisms for the alkalosis include increased renal secretion of bicarbonate, potassium, sodium and water, alveolar hypoventilation with an increase in blood carbon dioxide concentration and a shift of hydrogen ions from the intracellular space to the extracellular space. Once these compensatory mechanisms are exhausted, the blood pH increases along with a base excess. The increased renal excretion of water causes dehydration, haemoconcentration and azotaemia. Hypokalaemia results from renal potassium excretion as well as the movement of potassium from the blood into the intracellular space to compensate for the intracellular loss of hydrogen ions. Hypokalaemia is further enhanced by a lack of forage intake [31] accompanied by a relatively high milk yield in some cows [32]. In summary, these changes result in a hypochloraemic, hypokalaemic metabolic alkalosis accompanied by dehydration and azotaemia.

Ultrasonographic examination of cows with LDA from the left side shows that the abomasum is located between the abdominal wall and rumen [20–22, 33]. Ventrally, hypoechogenic to echogenic ingesta, often interspersed with crescent-shaped echogenic abomasal folds can be seen. Dorsally, the abomasal gas cap is characterised by reverberation artifacts, which appear as a series of equally spaced lines that run parallel to the transducer. The ultrasonographic findings in cows with RDA and AV obtained from the right side are nearly identical to those from cows with LDA [23]; in the last intercostal spaces, the displaced abomasum is situated between the right abdominal wall and the liver. Ultrasonographic differentiation of RDA and AV is not reliable [23, 33].

Although LDA, RDA and AV are well-known disorders of cattle, a comparative evaluation of the clinical, laboratory and ultrasonographic findings of these types of abomasal displacements has not been undertaken. Therefore, the objective of this study was to compare these findings in a large population of cows. A secondary goal was to determine the proportion of cases in which the definitive diagnosis was achieved using the results of clinical examination, ultrasonography or exploratory surgery.

## Methods

From a dissertation that comprised 2043 data sets [34], 61 records were excluded because the ultrasonographic findings had been previously published [21, 23]. Finally, the medical records of 1982 cows (2043–61 = 1982) referred to the Department of Farm Animals, University of Zurich, between January 1, 1988 and December 31, 2016 with a diagnosis of LDA (n = 1341), RDA (n = 338) and AV (n = 303) were analysed. All cows underwent the same structured clinical and laboratory examination procedures conducted by, or under the supervision of, the first author.

### Inclusion and exclusion criteria

Only dairy cows with an LDA, RDA or AV at the time of referral and in which the diagnosis was confirmed unequivocally during laparotomy or postmortem examination were included. Another 423 cattle were excluded before the start of the dissertation because they were calves (n = 60), juvenile heifers (n = 53), bulls (n = 9) and beef cows (n = 3). Fourteen other cows were excluded because the diagnosis of LDA was tentative, and others were excluded because abomasal displacement only became manifest during hospitalisation (n = 50), the cows had a recurring displacement (n = 70) or there was a pendulous abomasum (n = 139). Fourteen cows were excluded because they had another underlying disease, for instance a mechanical ileus with secondary dilation and displacement of the abomasum. The records of ten cows with insufficient data and the record of a previously published case of LDA and *situs inversus* [35] were also excluded. The final number of cases included in the study was 1982.

### Cows

All cows had been referred to the Veterinary Teaching Hospital, University of Zürich, by practicing veterinarians for diagnostic workup and treatment. Most cows originated from the Swiss Central Plateau but some were from the adjacent prealpine regions of the eastern, north-eastern and central parts of Switzerland. The cows were 3.0 to 6.0 years (25th to 75th percentiles) of age (mean ± sd = 4.7 ± 1.9 years) and belonged to the Holstein (n = 1155; 58.3%), Swiss Fleckvieh (n = 639, 32.2%), Brown Swiss (n = 146, 7.4%) and other dairy breeds (n = 42, 2.1%) (Table 1). Of all cows, 81.5% (n = 1426) were within 28 days in milk, 88.3% (n = 1730) were open or less than 5 weeks pregnant, 7.7% (n = 150) were 2 to 7 months pregnant and 4.1% (n = 80) were 8 to 9 months pregnant. At the time of admission, the duration of illness had ranged from 2 to 5 days (25th to 75th percentiles); on average, it was significantly shorter in cows with RDA (3.0 days) and AV (2.8 days) than in cows with LDA (4.5 days) (P < 0.05). 36.2% of cows with LDA had been ill for at least 5 days compared with only 17.1% of cows with RDA and 14.3% of cows with AV.

**Table 1:** Breed, age, stage of lactation and other findings in 1982 cows with LDA, RDA and AV

Variable	Finding	LDA	RDA	AV	Total	Chi^2^	Additional tests	P
Age (n = 1982)	Mean/median	4.7/4.6^a^	4.8/4.4^ab^	4.4/4.0^c^	4.7/4.4	NA	KW, BoCo	< 0.05
	SD (25–75th pc)	1.9 (3.0–6.0)	2.1(3.3–5.8)	1.8 (3.0–5.2)	1.9 (3.0–6.0)			
Breed (n = 1982)	Holstein	806^a^ (60.1%)	181^b^ (53.6%)	168^ab^ (55.4%)	1155 (58.3%)	36^**^	Boph	< 0.05
	Swiss Fleckvieh	433 (32.3%)	104 (30.8%)	102 (33.7%)	639 (32.2%)			
	Brown Swiss	71^a^ (5.3%)	49^b^ (14.5%)	26^c^ (8.6%)	146 (7.4%)			
	Other	31 (2.3%)	4 (1.2%)	7 (2.3%)	42 (2.1%)			
Stage of lactation (n = 1750)	Weeks 1 and 2	628^a^ (50.7%)	155^a^ (57.8%)	95^b^ (38.9%)	878 (50.2%)	33^**^	Boph	< 0.05
	Weeks 3 and 4	405^a^ (32.7%)	66^b^ (24.6%)	77^ab^ (31.6%)	548 (31.3%)			
	> 4 weeks	205^a^ (16.6%)	47^a^ (17.5%)	72^b^ (29.5%)	324 (18.5%)			
Gestation stage (n = 1960)	Open or less than 5 weeks	1234^a^ (92.6%)	265^b^ (79.1%)	231^b^ (78.8%)	1730 (88.3%)	82^**^	Boph	< 0.05
	2–7 months	62^a^ (4.7%)	42^b^ (12.5%)	46^b^ (15.7%)	150 (7.7%)			
	8–9 months	36^a^ (2.7%)	28^b^ (8.4%)	16^b^ (5.5%)	80 (4.1%)			
Duration of illness	Mean/median	4.5/3.0^a^	3.0/2.0^b^	2.8/2.0^b^	3.9/3.0	NA	KW, BoCo	< 0.05
(days) (n = 1450)	SD (25–75th pc)	3.7 (2.0–7.0)	2.5 (1.0–3.0)	2.1 (1.0–4.0)	3.4 (2.0–5.0)			

*KW* Kruskal Wallis test, *BoCo* Bonferroni correction, Boph bonferroni post hoc test, *pc* percentiles, *NA* not applicable,

^**^P < 0.01, within rows, values with different superscripts differ (P < 0.05)

### Clinical examination

The cows underwent a thorough clinical examination [36]. General health was evaluated by determining demeanour, the appearance of the hair coat and muzzle, skin elasticity, the position of the eyes in the sockets and skin surface temperature. General health was classified as normal or mildly, moderately or severely abnormal. Cows with a normal health status were bright and alert and had normal behaviour, posture and appetite, and a severely abnormal health status was characterized by listlessness and complete anorexia. Each cow was observed for signs of pain such as spontaneous grunting and bruxism. The rumen was assessed for degree of fill, the number and intensity of contractions. Auscultation of the rumen was carried out at two locations on the left side: the costal part of the abdominal wall and the centre of the flank (so-called double auscultation) [21]). Double auscultation was considered negative when rumen sounds were heard in the flank but not over the ribs. Double auscultation could not be assessed in cows that had no rumen contractions because of rumen atony. Sensitivity in the reticular region was assessed by preventing the animal from breathing for a short period by placing a plastic rectal sleeve over the mouth and nose and listening for grunting during the ensuing deep breath. This was followed by foreign body tests, which included the pole test, back grip and percussion of the abdominal wall over the region of the reticulum using a rubber hammer. Each test was carried out four times as described [37], and the reaction of the animal was observed each time. A test was considered positive when it elicited a short grunt a minimum of three of four times. The response to a test was considered questionable when it elicited a grunt two of four times and negative when the animal did not grunt or grunted only once. BSA as well as PSA of the abdomen on both sides and rectal examination were also carried out. Faeces were assessed for colour, consistency, amount, fibre particle length and abnormal contents. Cows that had calved within seven days and cows with vaginal discharge or abnormal findings of transrectal uterine palpation underwent a vaginal examination (data not shown). A urine sample was collected for analysis in 1923 (97.0%) cows; this was achieved by spontaneous voiding or by voiding induced by stroking of the escutcheon. Bladder catheterisation was used only when the other methods failed.

### Laboratory analyses

The following blood samples were collected from all cattle immediately after the clinical examination: 5 mL of EDTA blood for haematological analysis, 10 mL of whole blood for serum biochemistry and 2 mL of whole blood mixed with 0.2 mL heparin for venous blood gas analysis. Haematological analysis included the determination of haematocrit, total leukocyte count and the concentrations of total protein and fibrinogen. The samples were analysed using the Contraves analyzer AL820 (Contraves, Oerlikon, Switzerland) or the CELL-DYN 3500 (Abbott Diagnostics Division, Baar, Switzerland). These two analysers generate equivalent results [38]. The concentrations of serum urea, calcium, inorganic phosphate, potassium, chloride, bilirubin, and the activities of the enzymes aspartate aminotransferase (AST), γ-glutamyltransferase (γ-GT), glutamate dehydrogenase (GLDH) and sorbit dehydrogenase (SDH) were determined at 37 °C using an automated analyzer (Cobas Mira, Cobas Integra 700, Cobas Integra 800, Roche Diagnostics, Basel, Switzerland) and the manufacturer’s reagents (Roche-Reagents) according to the International Federation of Clinical Chemistry and Laboratory Medicine (IFCC). The venous blood gas analysis was performed with the RapidLab 248 analyser (Siemens Schweiz AG, Zurich, Switzerland). All haematology analysers used in the present study were equipped with a multi-species software that included the bovine species. Prior to use in patient/study samples the analyser was internally validated for its accuracy and precision in evaluating bovine blood. Haematocrit values of the instrument were adjusted to align with manually centrifuged PCV values, and non-statistical quality control was done by checking histograms and scatterplots. Haematology and chemistry analysers underwent statistical quality control daily on two levels using quality control materials. Proficiency testing was also carried out four times per year. Urine samples were analysed using a test strip (Combur^9^, Roche, Basel, Switzerland), and the urine specific gravity was determined using a refractometer (Krüss Optronic, Hamburg, Germany). A sample of rumen fluid was collected using a Dirksen probe and assessed for colour, odour, consistency and pH (data not shown). In addition, the concentration of chloride was determined (MK-II-Chlorid-Analyser 9265, Sherwood, Cambridge, Great Britain).

### Abdominal ultrasonography

Abdominal ultrasonography was done in 934 cows with LDA and 504 cows with RDA/AV immediately after the clinical examination and collection of blood. The last three intercostal spaces on the left side and the last five  intercostal spaces and the flank on the right side were scanned [33]. The ultrasound machines had the following 3.5- and 5.0 MHz transducers: An LSC 7000, Picker International GmbH, 3.5 MHz linear transducer was used from 1988 to 1996; Hitachi EUB-515A, Hitachi EUB 6000, Hitachi EUB 8500 and Ecoscan Ultrasound Holding 3.5 MHz transducers were used from 1997 to 2009; and a 5.0 MHz convex transducer from GE Medical Systems (Logiq 7, Logiq 9) was used from 2010 to 2016.

### Concomitant diseases

The type and number of comorbidities had been recorded for each cow. The clinical and laboratory variables of cows with one, two, three or more comorbidities and those of cows with no comorbidities were compared.

### Diagnosis

The gold standard for diagnosis of LDA, RDA and AV was laparotomy findings in operated cattle and postmortem findings in cattle that were euthanised. A clinical diagnosis of LDA was made when BSA and/or PSA were positive on the left side and double auscultation of the rumen was negative (rumen contractions were heard in the flank but were absent or muffled during auscultation over the ribs). An ultrasonographic diagnosis of LDA was made when the abomasum was visualised between the rumen and the left costal abdominal wall [33].

The clinical diagnosis of RDA or AV was made when BSA and/or PSA were positive on the right side and there were no signs of small or large intestinal ileus such as dilated intestines detected during transrectal palpation. The diagnosis was supported when the dilated abomasum could be palpated transrectally or when spontaneous abomasal sounds could be heard at the level of the displaced abomasum. The ultrasonographic diagnosis of RDA/AV was made when the dilated and displaced abomasum was visualised between the liver and the right costal abdominal wall [23, 33]. Clinical and ultrasonographic differentiation of RDA and AV was not possible. The final diagnosis was made during laparotomy and/or postmortem examination.

### Statistical analysis

Each cow was assigned to either the LDA, RDA or AV group. The clinical, laboratory and ultrasonographic variables were analysed for all cows combined and for each type of displacement separately. The data were compiled using FileMaker Pro Advanced 13.0 (File-Maker Inc., Santa Clara, CA, USA) and analysed with IBM^®^ SPSS^®^ Statistics 27.0 (IBM Corp. 2015, USA). The frequency distribution was determined for each variable. The Shapiro–Wilk test and the Kolmogorov–Smirnov test were used to test the data for normality. Numerical variables are presented as means ± standard deviations and medians, and the 25th and 75th percentiles were calculated. Differences between numerical data of the different types of displacement were analysed using the Kruskal–Wallis test; when the P-value was < 0.05, a Bonferroni correction was done to determine which groups differed with regard to the various variables. Differences between nominal data were analysed using the chi-square test. When differences were significant, a Bonferroni post hoc test for multiple comparisons was carried out to determine the diseases that differed with regard to the analysed nominal variables. For each variable, the diagnostic sensitivity (a/[a + c])was calculated (a, true positive; c, false negative;)[39].^1^ A true positive result was a normal finding and a false negative result was an abnormal finding in a cow with the disease. Pearson correlation coefficients were calculated for metric variables (heart and respiratory rates, rectal temperature). Correlation coefficients were also calculated for all laboratory variables. A P-value < 0.05 was considered significant.

## Results

The Shapiro–Wilk and Kolmogorov–Smirnov tests showed that all numerical variables had a non-normal distribution.

### Demeanour, posture, abdomen, signs of pain

The demeanour was mildly abnormal in 51.2% of the cows, moderately abnormal in 43.6% and severely abnormal in 4.6% (Table 2). Moderate or severe impairment of health was significantly more common in cows with RDA and AV compared with cows with LDA (P < 0.01).

**Table 2:** Demeanour, appearance and signs of pain in 1982 cows with LDA, RDA and AV

Variable	Finding	LDA n (%)	RDA n (%)	AV n (%)	Total n (%)	Chi^2^	Additional tests	P
Demeanour	Normal	8 (0.6)	1 (0.3)	2 (0.7)	11 (0.6)	189^**^	Boph	< 0.05
	Mildly abnormal	805^a^ (60.0)	139^b^ (41.1)	71^c^ (23.4)	1015 (51.2)			
	Moderately abnormal	500^a^ (37.3)	176^b^ (52.1)	189^c^ (62.4)	865 (43.6)			
	Severely abnormal	28^a^ (2.1)	22^b^ (6.5)	41^c^ (13.5)	91 (4.6)			
Posture	Arched back	76 (5.7)	17 (5.0)	12 (4.0)	105 (5.3)	1.5^ ns^	–	–
	Sawhorse stance	31^a^ (2.3)	22^b^ (6.5)	22^b^ (7.3)	75 (3.8)	25^**^	Boph	< 0.05
	Abducted elbows	33 (2.5)	8 (2.4)	5 (1.7)	46 (2.3)	0.7^ ns^	–	–
	Droopy ears	15^a^ (1.1)	13^b^ (3.8)	13^b^ (4.3)	41 (2.1)	19^**^	–	–
	Head and neck extended	8 (0.6)	2 (0.6)	2 (0.7)	12 (0.6)	0.1^ ns^	–	–
	Low head carriage	6 (0.4)	1 (0.3)	2 (0.7)	9 (0.5)	0.5^ ns^	–	–
	Recumbent	10 (0.7)	5 (1.5)	5 (1.7)	20 (1.0)	2.9^ ns^	–	–
Abdomen	Normal	1042^a^ (77.7)	293^b^ (86.7)	225^a^ (74.3)	1560 (78.7)	386^**^	Boph	< 0.05
	Left flank distended	281^a^ (21.0)	0^b^ (0)	0^b^ (0)	281 (14.2)			
	Right flank distended	0^a^ (0)	30^b^ (8.9)	52^c^ (17.2)	82 (4.1)			
	Enlarged	15^a^ (1.1)	12^b^ (3.6)	24^c^ (7.9)	51 (2.6)			
	Papple shape	3 (0.2)	3 (0.9)	2 (0.7)	8 (0.4)			
Signs of pain	Bruxism	209 (15.6)	45 (13.3)	38 (12.5)	292 (14.7)	2.5^ ns^	–	–
	Shifting weight in hind limbs	56^a^ (4.2)	19^ab^ (5.6)	23^b^ (7.6)	98 (4.9)	6.5^*^	Boph	< 0.05
	Muscle tremors	44 (3.3)	14 (4.1)	13 (4.3)	71 (3.6)	1.1^ ns^	–	–
	Colic	9^a^ (0.7)	23^b^ (6.8)	25^b^ (8.3)	57 (2.9)	73^**^	Boph	< 0.05
	Spontaneous grunting	18^a^ (1.3)	8^ab^ (2.4)	12^b^ (4.0)	38 (1.9)	9.4^**^	Boph	< 0.05

Boph Bonferroni post hoc test

^*Ns*^ not significant

^*^P < 0.05

^**^P < 0.01

Within rows, values with different superscripts differ (P < 0.05)

Abnormal posture manifested as an arched back (5.3%), sawhorse^2^ stance (3.8%), lateral abduction of the elbows (2.3%), droopy ears (2.1%), extended head and neck (0.6%) and lowered head carriage (0.5%). Sawhorse stance and droopy ears were more common in cows with RDA or AV compared with cows with LDA (P < 0.05). At admission, 1.0% of the cows were recumbent.

Distention of the left flank was obvious in 21.0% of the cows with LDA, and distention of the right flank occurred in 17.2% of cows with AV and 8.9% of cows with RDA (P < 0.05). Cows with RDA or AV had abdominal enlargement more often than cows with LDA (3.6 and 7.9% vs. 1.1%) (P < 0.01). A papple-shaped abdomen^3^ as viewed from behind was apparent in 0.4% of all cows. The most common sign of pain was bruxism (14.7%), followed by weight shifting in the hind legs (4.9%), muscle tremors (3.6%), signs of colic (2.9%) and spontaneous grunting (1.9%). Weight shifting was more common in cows with AV than in cows with LDA (7.6% vs. 4.2%) (P < 0.05) as was spontaneous grunting (4.0% vs. 1.3%) (P < 0.05), and signs of colic were more common in cows with AV and RDA than in cows with LDA (8.3% and 6.8% vs. 0.7%) (P < 0.01).

### Heart and respiratory rates and rectal temperature

The heart rate (reference interval, 60–80 bpm) ranged from 36 to 160 bpm, and cows with AV had a higher heart rate than cows with LDA and RDA (91 vs. 84 bpm) (P < 0.01) (Table 3). The heart rate was within the reference interval (60 to 80 bpm) in 46.7% of all cows, decreased (36 to 59 bpm) in 2.6% and increased (81 to 160 bpm) in 50.6%. The percentage of cows with severe tachycardia (121 to 160 bpm) was higher in cows with AV and RDA than in cows with LDA (6.3% and 4.1% vs. 1.7%) (P < 0.01).

**Table 3:** Heart rate, respiratory rate and rectal temperature in cows with LDA, RDA and AV (means, medians, standard deviations, 25th to 75th percentiles, frequency distributions)

Variable	Finding	LDA	RDA	AV	Total	Chi^2^	Additional tests	P
Heart rate	Mean/Median	84/80^a^	84/80^a^	91/88^b^	85/84	NA	KW, BoCo	< 0.05
(beats/min) (n = 1981)	SD (25–75th pc)	16 (72–92)	19 (72–96)	20 (76–104)	17 (72–96)			
	Normal (60–80)	663^a^ (49.5%)	162^a^ (47.9%)	101^b^ (33.3%)	926 (46.7%)	67**	Boph	< 0.05
	Decreased (36–59)	31 (2.3%)	12 (3.6%)	9 (3.0%)	52 (2.6%)			
	Mildly increased (81–100)	497 (37.1%)	116 (34.3%)	110 (36.3%)	723 (36.5%)			
	Moderately increased (101–120)	126^a^ (9.4%)	34^a^ (10.1%)	64^b^ (21.1%)	224 (11.3%)			
	Severely increased (121–160)	23^a^ (1.7%)	14^b^ (4.1%)	19^b^ (6.3%)	56 (2.8%)			
Respiratory rate	Mean/Median	30/28	30/28	29/28	30/28	NA	KW	> 0.05
(breaths/min)	SD (25–75th pc)	13 (20–36)	13 (20–36)	11 (22–36)	13 (20–36)			
(n = 1976)	Normal (15–25)	603 (45.1%)	142 (42.1%)	140 (46.5%)	885 (44.8%)	4.6	-	-
	Decreased (12–14)	21 (1.6%)	7 (2.1%)	6 (2.0%)	34 (1.7%)			
	Mildly increased (26–35)	359 (26.8%)	100 (29.7%)	76 (25.2%)	535 (27.1%)			
	Moderately increased (36–45)	223 (16.7%)	62 (18.4%)	53 (17.6%)	338 (17.1%)			
	Severely increased (46–120)	132 (9.9%)	26 (7.7%)	26 (8.6%)	184 (9.3%)			
Rectal temperature	Mean/Median	39.0/39.0 ^a^	38.9/38.9 ^ab^	38.7/38.8 ^b^	38.9/38.9	NA	KW, BoCo	< 0.05
(°C) (n = 1969)	SD (25–75th pc)	0.5 (38.6–39.3)	0.6 (38.5–39.2)	0.7 (38.3–39.2)	0.6 (38.6–39.2)			
	Normal (38.5–38.9)	444 (33.2%)	104 (31.0%)	82 (27.6%)	630 (32.0%)	46**	Boph	< 0.05
	Decreased (36.1–38.4)	209^a^ (15.6%)	79^b^ (23.5%)	93^b^ (31.3%)	381 (19.3%)			
	Mildly increased (39.0–39.4)	492 (36.8%)	105 (31.3%)	93 (31.3%)	690 (35.0%)			
	Moderately increased (39.5–40.0)	161 (12.1%)	37 (11.0%)	25 (8.4%)	223 (11.3%)			
	Severely increased (40.1–41.8)	30 (2.2%)	11 (3.3%)	4 (1.3%)	45 (2.3%)			

Within rows, values with different superscripts differ (P < 0.05)

*KW* Kruskal Wallis test, *BoCo* Bonferroni correction, Boph Bonferroni post hoc test, *pc* percentiles, *NA* not applicable, *SD* standard deviation

^**^P < 0.01

The respiratory rate (reference interval, 15–25 breaths/min) ranged from 12 to 120 breaths per min with a mean of 30 breaths per min. It was within the reference interval (15 to 25 breaths per min) in 44.8% of all cows, decreased (12 to 14 breaths per min) in 1.7% and increased (26 to 120 breaths per min) in 53.5%. The respiratory rate did not differ significantly among the three groups.

The rectal temperature ranged from 36.1 to 41.8 °C and was within the reference interval (38.5 to 38.9 °C) in 32.0% of all cows, decreased (36.1 to 38.4 °C) in 19.3% and increased (39.0–41.8 °C) in 48.6%. A decreased rectal temperature was more common in cows with RDA and AV than in cows with LDA (23.5% and 31.3% vs. 15.6%) (P < 0.01). The correlation coefficients r for heart and respiratory rates and rectal temperature ranged from 0.11 to 0.34 (P < 0.01) (heart rate x respiratory rate, r = 0.25; heart rate x rectal temperature, r = 0.11; respiratory rate x rectal temperature, r = 0.34).

### Gastrointestinal tract

Rumen contractions were reduced or absent in 89.7% of all cows (Table 4). Cows with LDA had rumen atony more often than cows with RDA (46.2% vs. 36.1%) (P < 0.05). Reduction in rumen fill occurred in 75.5% of all cows and was more common in cows with LDA than in cows with RDA and AV (79.7% vs. 71.5% and 61.9%) (P < 0.01). The rumen appeared to be fuller than normal in 2.3% of the cows, and rumen tympany occurred in 4.7%; these two findings were more common in cows with RDA and AV than in cows with LDA (P < 0.01). Double auscultation of the rumen was negative in 87.0% of cows with LDA, in 4.6% of cows with RDA and in 6.9% of cows with AV (P < 0.01).

**Table 4:** Gastrointestinal findings in cattle with LDA, RDA and AV

Variable	Finding	LDA n (%)	RDA n (%)	AV n (%)	Total	Chi^2^	Additional tests	P
Rumen motility (n = 1980)	Reduced	595 (44.4)	171 (50.6)	135 (44.6)	901 (45.5)	14	Boph	< 0.05
	Absent	619^a^ (46.2)	122^b^ (36.1)	135^ab^ (44.6)	876 (44.2)			
Rumen fill (n = 1962)	Reduced	1054^a^ (79.7)	241^b^ (71.5)	187^c^ (61.9)	1482 (75.5)	76^**^	Boph	< 0.05
	Fuller than normal	14^a^ (1.1)	13^b^ (3.9)	18^b^ (6.0)	45 (2.3)			
	Tympanic	40^a^ (3.0)	23^b^ (6.8)	30^b^ (9.9)	93 (4.7)			
Double auscultation of the rumen (n = 1418)	Negative	851^a^ (87.0)	11^b^ (4.6)	14^b^ (6.9)	876 (61.8)	466^**^	Boph	< 0.05
Positive foreign body tests^1^	Back grip (n = 1935)	121^a^ (9.2)	53^b^ (16.1)	29^ab^ (9.9)	203 (10.5)	14^**^	Boph	< 0.05
	Pole test (n = 1887)	108 (8.4)	20 (6.3)	24 (8.5)	152 (8.1)	3	-	-
	Percussion of the reticulum (n = 1928)	15 (1.1)	3 (0.9)	3 (1.0)	21 (1.1)	1.2	-	-
BSA and PSA on the left side (n = 1982)	Both negative (normal)	41^a^ (3.1)	330^b^ (97.6)	297^b^ (98.0)	668 (33.7)	1743^**^	Boph	< 0.05
	Only BSA positive	13 (1.0)	1 (0.3)	0 (0.0)	14 (0.7)			
	Only PSA positive	269^a^ (20.1)	3^b^ (0.9)	2^b^ (0.7)	274 (13.8)			
	Both positive	1017^a^ (75.8)	4^b^ (1.2)	4^b^ (1.3)	1025 (51.7)			
BSA and PSA on the right side (n = 1982)	Both negative (normal)	898^a^ (67.0)	5^b^ (1.5)	2^b^ (0.7)	905 (45.7)	1219^**^	Boph	< 0.05
	Only BSA positive	136^a^ (10.1)	5^b^ (1.5)	3^b^ (1.0)	144 (7.3)			
	Only PSA positive	124^a^ (9.3)	12^b^ (3.6)	2^c^ (0.7)	138 (7.0)			
	Both positive	182^a^ (13.6)	316^b^ (93.5)	296^b^ (97.7)	794 (40.1)			
Intestinal motility (n = 1950)	Reduced	645^a^ (48.9)	207^b^ (62.2)	171^b^ (57.6)	1023 (52.5)	177^**^	Boph	< 0.05
	Absent	56^a^ (4.2)	51^b^ (15.3)	60^b^ (20.2)	167 (8.6)			
Amount of faeces in the rectum (n = 1975)	Reduced	619^a^ (46.4)	221^b^ (65.4)	179^b^ (59.3)	1019 (51.6)	183^**^	Boph	< 0.05
	No faeces	77^a^ (5.8)	50^b^ (14.8)	63^c^ (20.9)	190 (9.6)			
Colour of faeces (n = 1982)	Dark brown to black	78^a^ (5.8)	58^b^ (17.2)	58^b^ (19.1)	194 (9.8)	83^**^	Boph	< 0.05
Consistency of faeces (n = 1982)	Thin porridge-like	252^a^ (18.8)	58^ab^ (17.2)	35^b^ (11.6)	345 (17.4)	35^**^	Boph	< 0.05
	Thick porridge-like	160 (11.9)	42 (12.4)	30 (9.9)	232 (11.7)			
	Watery	85^a^ (6.3)	32^ab^ (9.5)	33^b^ (10.9)	150 (7.6)			
	Pasty	58^a^ (4.3)	17^ab^ (5.0)	29^b^ (9.6)	104 (5.2)			
	Biphasic	19 (1.4)	7 (2.1)	9 (3.0)	35 (1.8)			
Degree of comminution (n = 1982)	Reduced	360^a^ (26.8)	69^b^ (20.4)	56^b^ (18.5)	485 (24.5)	17^**^	Boph	< 0.05
Abnormal contents (n = 1982)	Mucus	108^a^ (8.1)	42^b^ (12.4)	31^ab^ (10.2)	181 (9.1)	43^**^	Boph	< 0.05
	Blood	47^a^ (3.5)	24^b^ (7.1)	27^b^ (8.9)	98 (4.9)			
	Fibrin	16 (1.2)	4 (1.2)	7 (2.3)	27 (1.4)			
Rectal findings (n = 1982)	Abomasum palpable	15^a^ (1.1)	38^b^ (11.2)	67^c^ (22.1)	120 (6.1)	294	Boph	< 0.05

*BSA* ballottement and simultaneous auscultation, *PSA* percussion and simultaneous auscultation

Within rows, values with different superscripts differ (P < 0.05), Boph Bonferroni post hoc test

^1^Positive: at least 3 of 4 tests elicited a grunt

^**^ P < 0.01

Of the foreign body tests, positive results were seen with the back grip in 10.5% of cows, the pole test in 8.1% and percussion of the reticular area in 1.1%; a positive back grip test was more common in cows with RDA than in cows with LDA (16.1 vs. 9.2%) (P < 0.05). On the left side, BSA and/or PSA were positive in 96.9% of the cows with LDA and on the right, they were positive in 98.5% of the cows with RDA and in 99.3% of the cows with AV. Intestinal motility was reduced or absent in 61.1% of all cows; this finding was more common in cows with RDA and AV than in cows with LDA (77.5% and 77.8% vs. 53.1%) (P < 0.01). Compared with healthy cows, the amount of faeces in the rectum was decreased in 51.6% of all cows, and in 9.6%, the rectum was empty. In cows with AV, the latter finding was more common than in cows with LDA and RDA (20.9% vs. 5.8% and 14.8%) (P < 0.01). Cows with no intestinal motility had an empty rectum more often than cows with reduced or normal intestinal motility (25.7% vs. 10.4% and 4.8%) (P < 0.01). Dark or black manure was more common in cows with RDA and AV than in cows with LDA (17.2% and 19.1% vs. 5.8%) (P < 0.01). Thin porridge-like faeces occurred in 17.4% of all cows, thick porridge-like faeces in 11.7% and watery faeces in 7.6%. In 24.5% of all cows, the faeces contained poorly digested plant fragments. Abnormal faecal contents included mucus (9.1%), blood (4.9%) and fibrin (1.4%); cows with RDA had mucus in the faeces more often than cows with LDA (P < 0.01), and cows with RDA and AV had blood in the faeces more often than cows with LDA (P < 0.01). The results of transrectal palpation of the abomasum also differed among the three groups; the abomasum was palpated more frequently in cows with AV than in cows with RDA and LDA (22.1% vs. 11.2% and 1.1%) (P < 0.01).

### Other clinical findings

A cool skin surface temperature was more common in cows with AV than in cows with RDA and LDA (57.0% vs. 44.5% and 25.7%) (Table 5). The same comparison was seen for reduction in skin turgor (33.6% vs. 25.1% and 25.4%) (P < 0.01) and for enophthalmos (43.9% vs. 32.5% and 30.1%) (P < 0.01). By contrast, moderate or severe hyperaemia of the scleral vessels was seen in all three groups with a mean frequency of 35.4%. The muzzle was found to be dry more often in cows with RDA and AV than in cows with LDA (17.8 and 18.5% vs. 9.2%) (P < 0.01). Uraemic fetor was more common in cows with AV than in cows with LDA (27.4% vs. 17.6%) (P < 0.01). The oral mucosa was normal in 86.9% of all cows and was more often pale in cows with AV and RDA than in cows with LDA (10.3 and 8.1% vs. 3.5%) (P < 0.01). The oral mucosa was hyperaemic in 5.8%, cyanotic in 1.0%, washed-out in 0.9% and icteric in 0.2% of all the cows. A capillary refill time > 2 s was more common in cows with RDA and AV than in cows with LDA (34.5 and 35.6% vs. 22.5%) (P < 0.01), and abdominal guarding was more common in cows with AV than in cows with RDA and LDA (61.1% vs. 47.9 and 28.0%) (P < 0.01).

**Table 5:** Additional clinical findings in cows with LDA, RDA and AV

Variable	Finding	LDA n (%)	RDA n (%)	AV n (%)	Total	Chi^2^	Additional tests	P
Skin surface temperature (n = 1954)	Reduced	339^a^ (25.7)	149^b^ (44.5)	170^c^ (57.0)	658 (33.7)	132^**^	Boph	< 0.05
Skin turgor (n = 1971)	Moderately to severely reduced	340^a^ (25.4)	84^a^ (25.1)	101^b^ (33.6)	525 (26.6)	16^*^		
Eyes (n = 1982)	Moderate to severe enophthalmos	403^a^ (30.1)	110^a^ (32.5)	133^b^ (43.9)	646 (32.6)	29^**^	Boph	< 0.05
Sclera (n = 1982)	Moderately to severely injected	460 (34.3)	124 (36.7)	119 (39.3)	703 (35.4)	14 ^ns^	–	–
Muzzle (n = 1982)	Dry	123^a^ (9.2%)	60^b^ (17.8%)	56^b^ (18.5%)	239 (12.0)	40^**^	Boph	< 0.05
Breath (n = 1982)	Uraemic	236^a^ (17.6)	76^ab^ (22.5)	83^b^ (27.4)	395 (19.9)	17^ ns^	–	–
Mucous membranes (n = 1970)	Normal	1185^a^ (88.8)	287^ab^ (85.7)	239^b^ (79.7)	1711 (86.9)	35^**^	Boph	< 0.05
	Pale	47^a^ (3.5)	27^b^ (8.1)	31^b^ (10.3)	105 (5.3)			
	Hyperaemic	77 (5.8)	16 (4.8)	21 (7.0)	114 (5.8)			
	Cyanotic	11 (0.8)	3 (0.0)	5 (1.7)	19 (1.0)			
	Washed-out	12 (0.9)	1 (0.3)	4 (1.3)	17 (0.9)			
	Icteric	3 (0.2)	1 (0.3)	0 (0.0)	4 (0.2)			
Capillary refill time (n = 1962)	> 2 s	299^a^ (22.5)	116^b^ (34.5)	105^b^ (35.6)	520 (26.5)	36^**^	Boph	< 0.05
Abdominal wall (n = 1982)	Tense	376^a^ (28.0)	162^b^ (47.9)	185^c^ (61.1)	723 (36.5)	139^**^	Boph	< 0.05

Within rows, values with different superscripts differ (P < 0.05), Boph Bonferroni post hoc test

*ns* not significant (P > 0.05)

^*^P < 0.05

^**^P < 0.01

### Diagnostic sensitivity of clinical findings

Four of the 29 clinical finding categories listed in Tables 2, 3, 4, 5 had diagnostic sensitivities > 90%: abnormal demeanour (LDA 99.4%, RDA 99.7%, AV 99.3%), reduced or absent rumen motility (LDA 91.1%), positive BSA and/or PSA on the left side (LDA 96.9%) and positive BSA and/or PSA on the right side (RDA 98.5%, AV 99.3%).

### Urinalysis

Urine pH ranged from 4.0 to 9.0, and cows with LDA had a higher mean urine pH than cows with RDA and AV (7.3 vs. 7.1 and 6.8) (P < 0.01) (Table 6). Cows with AV had acidic urine more often than cows with RDA and LDA (49.1% vs. 38.6 and 33.0%) (P < 0.01). Urine specific gravity in cows with LDA was lower than in cows with RDA and AV (1.020 vs. 1.025 and 1.026) (P < 0.01), and cows with LDA had hyposthenuria more often than cows with RDA and AV (45.9% vs. 32.5 and 26.0%) (P < 0.01). Ketone bodies occurred in the urine of 49.0% of all cows, and cows with LDA had moderate to severe (+ +  + , +  +  + +) ketonuria more often than cows with RDA and AV (19.7% vs. 8.2 and 4.6%) (P < 0.01). Proteinuria was seen in 46.7% of all cows, haematuria in 45.4% and glucosuria in 21.1%. Moderate to severe (+ +  + , +  +  + +) haematuria and glucosuria were more common in cows with AV than in cows with LDA (P < 0.05).

**Table 6:** The results of urinalysis in cattle with LDA, RDA and AV (medians, 25th to 75th percentiles, frequency distributions)

Variable	Finding	LDA	RDA	AV	Total	Chi^2^	Additional tests	P
pH	Median	8.0^a^	7.0^b^	7.0^bc^	7.5	–	KW, BoCo	< 0.05
(n = 1905)	(25–75% pc)	(6.0–8.5)	(6.0–8.0)	(5.0–8.0)	1.4 (6.0–8.0)			
	Normal (7.0–8.0)	521 (40.1%)	126 (38.9%)	102 (36.3%)	749 (39.3%)	33^**^	Boph	< 0.05
	Decreased (4.0–6.9)	429^a^ (33.0%)	125^a^ (38.6%)	138^b^ (49.1%)	692 (36.3%)			
	Increased (8.1–9.0)	350^a^ (26.9%)	73^a^ (22.5%)	41^b^ (14.6%)	464 (24.4%)			
Specific gravity	Median	1.020^a^	1.025^b^	1.027^b^	1.022	–	KW, BoCo	< 0.05
(n = 1802)	(25–75% pc)	(1.010–1.028)	(1.015–1.032)	(1.018–1.035)	(1.012–1.030)			
	Normal (1.020–1.040)	622^a^ (50.8%)	179^b^ (58.7%)	178^b^ (65.2%)	979 (54.3%)	62^**^	Boph	< 0.05
	Decreased (1.000–1.039)	562^a^ (45.9%)	99^b^ (32.5%)	71^b^ (26.0%)	732 (40.6%)			
	Increased (1.041–1.060)	40^a^ (3.3%)	27^b^ (8.9%)	24^b^ (8.8%)	91 (5.0%)			
Ketone bodies (mg/dL)	Negative (< 10)	546^a^ (41.6%)	217^b^ (66.2%)	217^c^ (76.7%)	980 (51.0%)	164^**^	Boph	< 0.05
(n = 1923)	+ , + + (10–50)	507^a^ (38.6%)	84^b^ (25.6%)	53^c^ (18.8%)	644 (33.5%)			
	+ + + , + + + + (≥ 150)	259^a^ (19.7%)	27^b^ (8.2%)	13^b^ (4.6%)	299 (15.5%)			
Protein (mg/dL) (n = 1917)	Negative (< 30)	704 (53.8%)	166 (50.9%)	153 (54.1%)	1023 (53.4%)	2.6^ ns^	–	-
	+ , + + (30–100)	600 (45.9%)	159 (48.8%)	129 (45.6%)	888 (46.3%)			
	+ + + , + + + + (> 100)	4 (0.3%)	1 (0.3%)	1 (0.4%)	6 (0.4%)			
Erythrocytes (per high-	Negative (< 5)	737 (56.5%)	168 (51.7%)	138 (48.9%)	1043 (54.6%)	15^ ns^	–	–
power field) (n = 1912)	+ to + + (5–25)	354 (27.1%)	88 (27.0%)	75 (26.6%)	517 (27.0%)			
	+ + + , + + + + (50–250)	214 (16.4%)	69 (21.2%)	6 (24.5%)	352 (18.4%)			
Glucose (mg/dL)	Negative (< 50)	1093^a^ (83.4%)	242^b^ (74.2%)	178^c^ (63.1%)	1513 (78.8%)	80^**^	Boph	< 0.05
(n = 1919)	+ , + + (50–100)	145 (11.0%)	42 (12.8%)	53 (18.8%)	240 (12.5%)			
	+ + + , + + + + (300–1000)	73^a^ (5.5%)	42^b^ (12.9%)	51^b^ (18.1%)	166 (8.6%)			

*KW* Kruskal Wallis test, *BoCo* Bonferroni correction, Boph Bonferroni post hoc test, Within rows, values with different superscripts differ (P < 0.05). *ns* not significant, *pc* percentiles

^**^P < 0.01

### Ultrasonographic findings of the abomasum

Ultrasonography allowed correct identification of LDA significantly more often than RDA (P < 0.01, chi^2^ = 38.4). Findings characteristic of LDA were seen in 97.9% of 893 cows with LDA when examined on the left side [21]; the abomasum was visualised between the left abdominal wall and the rumen and contained heterogeneous echogenic ingesta and occasionally abomasal folds ventrally and a gas cap characterised by reverberation lines dorsally. The ultrasonographic findings could not be clearly interpreted in 19 (2.1%) cows. Of 489 cows with RDA or AV that were examined on the right side, findings typical of RDA/AV were obtained in 441 (90.2%) cows [21, 23] but differentiation of RDA and AV was not possible. The abomasum was situated between the right abdominal wall and the liver and its ultrasonographic appearance was similar to that of LDA. A final ultrasonographic diagnosis could not be made in 9.8% of the cows with RDA/AV.

### Laboratory findings

**Fig. 1:**
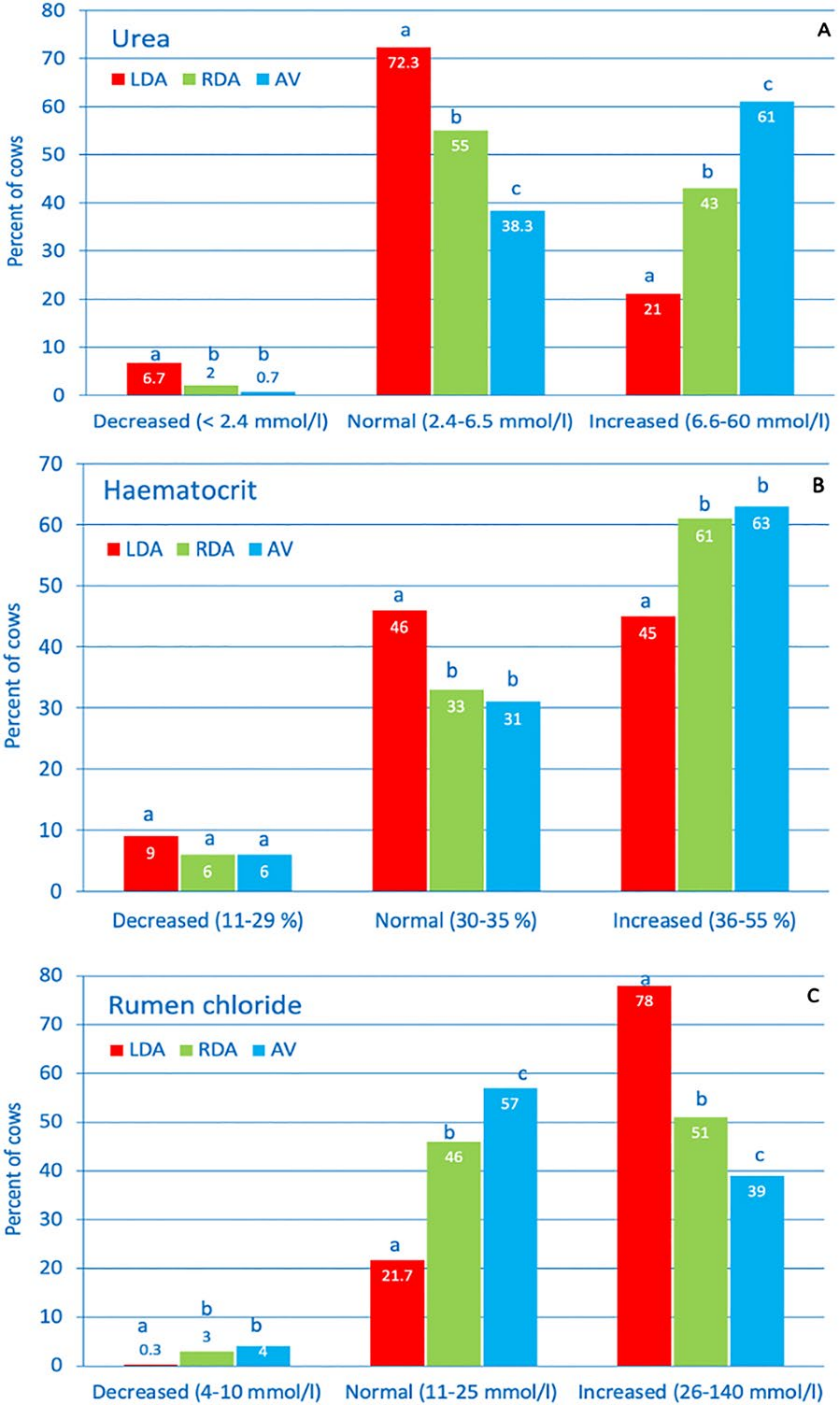
- Abnormal blood variables in cows with LDA, RDA and AV. Incidence of decreased, normal and increased values of blood urea concentration (**A**), haematocrit (**B**) and rumen chloride concentration (**C**) in cows with LDA, RDA and AV

The typical changes associated with abomasal reflux syndrome varied in severity. The rumen chloride concentrations differed significantly among the three groups (P < 0.01); the cows with LDA had the highest mean concentration (43 mmol/L) and the cows with AV had the lowest (Table 7). As a result of abomasal reflux of hydrochloric acid and ensuing compensatory mechanisms, 83% of cows had hypokalaemia, 67% had a base deficit, 50% had haemoconcentration, 45% had hypochloraemia, 40% had increased bicarbonate concentration in the blood and 31% had azotaemia (Table 8). The biggest differences among the three groups concerning the frequency of abnormal variables occurred with increased blood urea (chi^2^ = 212) (Table 8) and rumen chloride concentration (chi^2^ = 192) and haemoconcentration (chi^2^ = 52): the blood urea concentration was increased in 61% of the cows with AV compared with 43% of cows with RDA and 21% of cows with LDA (P < 0.01) (Fig. 1A). The comparison of haemoconcentration values was similar (AV 63%, RDA 61%, LDA 45%) (P < 0.01) (Fig. 1B). The rumen chloride concentration was increased in 78% of cows with LDA compared with 51% of cows with RDA and 39% of cows with AV (P < 0.01) (Fig. 1C).

**Table 7:** Laboratory findings in cattle with LDA, RDA and AV (medians, 25th to 75th percentiles)

Variable	Finding	LDA	RDA	AV	Total	Tests
Haematocrit (%)	Median	35^a^	37^b^	38^b^	36	KW, BoCo
	(25–75th pc)	(32–38)	(33–40)	(33–42)	(33–39)	
	n	1338	338	303	1979	
White blood cell	Median	7400^a^	7900^b^	9100^c^	7700	KW, BoCo
count (/µL)	(25–75th pc)	(5700–9800)	(6175–10,800)	(6850–12,250)	(5900–10,200)	
	n	1334	338	301	1973	
Total protein (g/L)	Median	76	74	77	76	KW
	(25–75th pc)	(70–82)	(68–81)	(70–84)	(70–82)	
	n	1329	337	302	1968	
Fibrinogen (g/L)	Median	6.0	6.0	6.0	6.0	KW
	(25–75th pc)	(4.0–7.0)	(4.0–7.0)	(4.0–8.0)	(4.0–7.0)	
	n	1326	337	302	1965	
Urea (mmol/L)	Median	4.8^a^	6.0^b^	7.9^c^	5.3	KW, BoCo
	(25–75th pc)	(3.6–6.2)	(4.6–8.5)	(5.4–12.3)	(4.0–7.2)	
	n	1335	336	303	1974	
Bilirubin (µmol/L)	Median	13.2^a^	11.7^b^	10.3^bc^	12.5	KW, BoCo
	(25–75th pc)	(9.3–18.0)	(7.6–16.8)	(6.7–15.7)	(8.7–17.5)	
	n	1329	332	301	1962	
AST (U/L)	Median	147^a^	191^b^	178^bc^	156	KW, BoCo
	(25–75th pc)	(108–216)	(125–310)	(126–280)	(113–235)	
	n	1336	336	303	1975	
γ-GT (U/L)	Median	31.0^a^	53.0^b^	65.0^bc^	36	KW, BoCo
	(25–75th pc)	(23–48)	(34–90)	(39–101)	(24–65)	
	n	1336	336	303	1975	
GLDH (U/L)	Median	52^a^	153^b^	148^bc^	71	KW, BoCo
	(25–75th pc)	(29–115)	(63–303)	(71–275)	(33–176)	
	n	967	249	224	1440	
SDH (U/L)	Median	32^a^	80^b^	76^bc^	42	KW, BoCo
	(25–75th pc)	(19–61)	(40–179)	(38–183)	(21–89)	
	n	958	247	217	1422	
Calcium (mmol/L)	Median	2.1^a^	2.1^ab^	2.2^c^	2.1	KW, BoCo
	(25–75th pc)	(2.0–2.3)	(1.9–2.2)	(2.1–2.3)	(2.0–2.3)	
	n	1111	265	232	1608	
Inorg. phosphate	Median	1.2^a^	1.4^b^	1.6^bc^	1.3	KW, BoCo
(mmol/L)	(25–75th pc)	(0.9–1.6)	(1.0–1.9)	(1.2–2.0)	(1.0–1.7)	
	n	1113	266	231	1610	
Potassium	Median	3.4^a^	3.3^ab^	3.1^c^	3.3	KW, BoCo
(mmol/L)	(25–75th pc)	(2.8–3.8)	(2.9–3.7)	(2.7–3.7)	(2.8–3.8)	
	n	1334	335	303	1972	
Chloride	Median	96^a^	97^ab^	93^c^	96	KW, BoCo
(mmol/L)	(25–75th pc)	(90–102)	(90–102)	(84–100)	(89–101)	
	n	1332	336	302	1970	
pH	Median	7.43^a^	7.41^b^	7.42^ab^	7.43	KW, BoCo
	(25–75th pc)	(7.39–7.47)	(7.37–7.46)	(7.38–7.46)	(7.39–7.47)	
	n	1224	312	276	1812	
Base excess	Median	5.1^a^	3.9^b^	5.0^ac^	4.8	KW, BoCo
(mmol/L)	(25–75th pc)	(0.8–10.2)	(-0.1–9.1)	(0.8–10.0)	(0.5–10.0)	
	n	1220	310	280	1810	
Bicarbonate	Median	29^a^	28^b^	29^ac^	28	KW, BoCo
(mmol/L)	(25–75th pc)	(25–34)	(24–33)	(25–33)	(24–33)	
	n	1224	313	280	1817	
pCO_2_	Median	46^a^	45^ab^	46^bc^	46	KW, BoCo
(mmHg)	(25–75th pc)	(35–41)	(37–42)	(38–43)	(42–51)	
	n	1225	311	276	1812	
L-lactate	Median	1.3^a^	1.9^b^	5.1^c^	1.7	KW, BoCo
(mmol/L)	(25–75th pc	(0.9–2.1)	(1.2–4.5)	(2.5–8.0)	(1.0–4.2)	
	n	115	47	47	209	
Rumen chloride	Median	41^a^	26^b^	23^c^	34	KW, BoCo
(mmol/L)	(25–75th pc)	(27–56)	(19–37)	(17–33)	(22–51)	
	n	1188	302	271	1761	

Within rows, values with different superscripts differ (P < 0.05)

*KW* Kruskal Wallis test, *BoCo* Bonferroni correction, pc percentiles

**Table 8:** Frequencies of abnormal laboratory findings in cows with LDA, RDA and AV

Variable	Finding	LDA	RDA	AV	Total	Chi^2^	Additional tests	P
Haematocrit (n = 1979)	≤ 35% > 35%	740 (55%) 598^a^ (**45%**)^1^	133 (39%) 205 (**61%**) ^b^	111 (37%) 192 (**63%**) ^b^	984 (50%) 995 (**50%**)	52^**^	Boph	< 0.05
Leukocytes (n = 1973)	≤ 10,000/µL > 10,000/µL	1041 (78%) 293 (**22%**)^a^	244 (72%) 94 (**28%**)^a^	181 (60%) 120 (**40%**)^b^	1466 (74%) 507 (**26%**)	42^**^	Boph	< 0.05
Total protein (n = 1968)	≤ 80 g/L > 80 g/L	942 (71%) 387 (**29%**)^a^	251 (74%) 86 (**26%**)^a^	201 (67%) 101 (**33%**)^a^	1394 (71%) 574 (**29%**)	4.9^ ns^	–	–
Fibrinogen (n = 1965)	≤ 7 g/L > 7 g/L	1018 (77%) 308 (**23%**)^a^	257 (76%) 80 (**24%**)^a^	222 (73%) 80 (**27%**)^a^	1497 (76%) 468 (**24%**)	1.4^ ns^	–	–
Urea (n = 1974)	≤ 6.5 mmol/L > 6.5 mmol/L	1055 (79%) 280 (**21%**)^a^	193 (57%) 143 (**43%**)^b^	118 (39%) 185 (**61%**)^c^	1366 (69%) 608 (**31%**)	212^**^	Boph	< 0.05
Calcium (n = 1608)	< 2.20 mmol/L ≥ 2.20 mmol/L	873 (**79%**)^ab^ 238 (21%)	221 (**83%**)^b^ 44 (17%)	167 (**72%**)^a^ 65 (28%)	1261 (**78%**) 347 (22%)	10^**^	Boph	< 0.05
Inorg. phosph. (n = 1610)	< 1.30 mmol/L ≥ 1.30 mmol/L	608 (**55%**)^a^ 504 (45%)	114 (**43%**)^b^ 152 (57%)	73 (**32%**)^c^ 158 (68%)	795 (**49%**) 814 (51%)	47^**^	Boph	< 0.05
Potassium (n = 1972)	< 4.0 mmol/L ≥ 4.0 mmol/L	1097 (**82%**) 237 (18%)	282 (**84%**) 53 (16%)	261 (**86%**) 42 (14%)	1640 (**83%**) 332 (17%)	3^ ns^	–	–
Chloride (n = 1970)	< 95 mmol/L ≥ 95 mmol/L	575 (**43%**)^a^ 757 (57%)	129 (**38%**)^a^ 207 (62%)	173 (**57%**)^b^ 129 (43%)	877 (**45%**) 1093 (55%)	26^**^	Boph	< 0.05
Bilirubin (n = 1962)	≤ 6.5 µmol/L > 6.5 µmol/L	141 (11%) 1188 (**89%**)^a^	62 (19%) 270 (**81%**)^b^	70 (23%) 231 (**77%**)^b^	273 (14%) 1689 (**86%**)	40^**^	Boph	< 0.05
AST (n = 1975)	≤ 103 U/L > 103 U/L	289 (22%) 1047 (**78%**)^a^	52 (15%) 284 (**85%**)^b^	37 (12%) 266 (**88%**)^b^	378 (19%) 1597 (**81%**)	18^**^	Boph	< 0.05
γ-GT (n = 1975)	≤ 30 U/L > 30 U/L	645 (48%) 691 (**52%**)^a^	70 (21%) 266 (**79%**)^b^	48 (16%) 255 (**84%**)^b^	763 (39%) 1212 (**61%**)	164^**^	Boph	< 0.05
γ-GT (n = 1975)	≤ 30 U/L > 30 U/L	645 (48%) 691 (**52%**)^a^	70 (21%) 266 (**79%**)^b^	48 (16%) 255 (**84%**)^b^	763 (39%) 1212 (**61%**)	164^**^	Boph	< 0.05
GLDH (n = 1440)	≤ 25 U/L > 25 U/L	198 (20%) 769 (**80%**)^a^	21 (8%) 228 (**92%**)^b^	11 (5%) 213 (**95%**)^b^	230 (16%) 1210 (**84%**)	46^**^	Boph	< 0.05
SDH (n = 1422)	≤ 7.6 U/L > 7.6 U/L	20 (2%) 938 (**98%**)	7 (3%) 240 (**97%**)	7 (3%) 210 (**97%**)	34 (2%) 1388 (**98%**)	2^ ns^	–	–
Blood pH	< 7.41	363 (**30%**)^a^	123 (**39%**)^b^	96 (**35%**)^ab^	582 (**32%**)	12^**^	Boph	< 0.05
(n = 1812)	> 7.45	445 (**36%**)	94 (**30%**)	95 (**34%**)	634 (**35%**)			
Bicarbonate (n = 1817)	< 20.0 mmol/L > 30.0 mmol/L	76 (**6%**) 496 (**41%**) ^ab^	13 (**4%**) 107 (**34%**) ^b^	16 (**6%**) 123 (**44%**)^a^	105 (**6%**) 726 (**40%**)	10^**^	Boph	< 0.05
Base excess	< -2.0 mmol/L	160 (**13%**)	39 (**13%**)	31 (**11%**)	230 (**13%**)	10^**^	Boph	< 0.05
(n = 1810)	> + 2.0 mmol/L	829 (**68%**)^a^	188 (**61%**)^b^	187(**67%**)^ab^	1204 (**67%**)			
L-lactate (n = 209)	≤ 2.2 mmol/L > 2.2 mmol/L	89 (77%) 26 (**23%**)^a^	27 (57%) 20 (**43%**)^b^	10 (21%) 37 (**79%**)^c^	126 (60%) 83 (**40%**)	45^**^	Boph	< 0.05
Rumen chloride (n = 1761)	≤ 25.0 mmol/L > 25.0 mmol/L	263 (22%) 925 (**78%**)^a^	148 (49%) 154 (**51%**)^b^	164 (61%) 107 **(39%**)^c^	575 (33%) 1186 (**67%**)	192^**^	Boph	< 0.05

Within rows, values with different superscripts differ (P < 0.05), Boph Bonferroni post hoc test

ns not significant

Bold values represent the diagnostic sensitivity (a/[a + b]) (true positives identified by the test as positive)

^*^ P < 0.05

^**^P < 0.01

Of all laboratory variables, only the activities of GLDH (RDA 92%, AV 95%) and SDH (LDA 98%, RDA 97%, AV 97%) had diagnostic sensitivities > 90% (Table 8). Numerous variables were significantly correlated (P < 0.01) but correlation coefficients > 0.80 were calculated only for blood gas variables (pH x bicarbonate, r = 0.81; pH x BE, r = 0.83; BE x bicarbonate, r = 0.99).

### Comorbidities

Of 1979 cows that were evaluated, 1729 (87.4%) had at least one comorbidity; 624 (31.5%) had one, 589 (29.8%) had two, 326 (16.5%) had three and 190 (9.6%) had more than three. The most common comorbidity was ketosis (36.8%, n = 728) followed by mastitis (31.5%, n = 624), metritis/endometritis (27.0%, n = 534) and hepatic lipidosis (21.7%, n = 429). Other accompanying diseases included gastrointestinal parasitism (11.6%, n = 230), abomasal ulcer (8.6%, n = 171), claw disorders (8.6%, n = 170), bronchopneumonia (3.4%, n = 67), fascioliasis (2.1%, n = 41) and caecal dilatation (1.1%, n = 21).

The clinical findings did not differ significantly between cows without and with one, two, three or more than three comorbidities. This applied to the mean respiratory and heart rates, rectal temperature and to the frequency distributions of all variables listed in Tables 2, 4, 5.

Laboratory variables did not differ significantly between cows with one and no concomitant diseases (Table 9). Cows with two comorbidities had one variable (bilirubin), cows with three comorbidities had three variables (urea, bilirubin, AST) and cows with four to eight comorbidities had six variables (urea, bilirubin, AST, γ-GT, bicarbonate, base excess) that were significantly increased (bilirubin, AST, γ-GT) or decreased (urea, bicarbonate, base excess) (Table 9). Despite the statistical significance, the differences were numerically small.

**Table 9:** Number of comorbidities and laboratory values (means) in cows with LDA, RDA and AV

Variable	Number of comorbidities					Additional tests	P
	None	One	Two	Three	Four to eight		
Haematocrit (%) (n = 1979)	37 (n = 250)	36 (n = 624)	36 (n = 589)	36 (n = 326)	35^a^ (n = 190)	BoCo	< 0.05
Leukocytes (/µL) (n = 1973)	9116 (n = 249)	8680 (n = 623)	8688 (n = 587)	8311^a^ (n = 325)	8284^a^ (n = 189)	BoCo	< 0.05
Total protein (g/L) (n = 1968)	77 (n = 250)	76 (n = 620)	77 (n = 583)	76 (n = 326)	75 (n = 189)	–	–
Fibrinogen (g/L) (n = 1965)	5.5 (n = 249)	5.7 (n = 618)	5.7 (n = 583)	5.7 (n = 326)	6.0 (n = 189)	–	–
Urea (mmol/L) (n = 1974)	7.3 (n = 247)	6.6 (n = 623)	6.5 (n = 588)	5.7^a^ (n = 326)	5.6^a^ (n = 190)	–	–
Calcium (mmol/L) (n = 1608)	2.2 (n = 186)	2.1 (n = 485)	2.1 (n = 490)	2.1 (n = 283)	2.1 (n = 164)	–	–
Inorg. phosphate (mmol/L) (n = 1610)	1.4 (n = 187)	1.4 (n = 487)	1.4 (n = 489)	1.4 (n = 283)	1.3 (n = 164)	–	–
Potassium (mmol/L) (n = 1972)	3.4 (n = 246)	3.4 (n = 623)	3.3 (n = 587)	3.4 (n = 326)	3.4 (n = 190)	–	–
Chloride (mmol/L) (n = 1970)	94 (n = 247)	95 (n = 623)	94 (n = 586)	95 (n = 324)	96^b^ (n = 190)	BoCo	< 0.05
Bilirubin (µmol/L) (n = 1962)	11.8 (n = 242)	13.0 (n = 619)	14.3^a^ (n = 587)	16.4^a^ (n = 324)	16.9 (n = 190)	–	–
AST (U/L) (n = 1975)	180 (n = 248)	198 (n = 622)	199 (n = 589)	235^a^ (n = 326)	247^a^ (n = 190)	–	–
γ-GT (U/L) (n = 1975)	51 (n = 248)	53 (n = 623)	64 (n = 588)	63 (n = 326)	75 (n = 190)	–	–
GLDH (U/L) (n = 1440)	135 (n = 140)	144 (n = 430)	151 (n = 448)	143 (n = 264)	157 (n = 158)	–	–
SDH (U/L) (n = 1422)	97 (n = 139)	98 (n = 426)	85 (n = 439)	82 (n = 262)	89 (n = 156)	–	–
Blood pH (n = 1812)	7.42 (n = 221)	7.42 (n = 558)	7.43 ( = 545)	7.42 (n = 312)	7.42 (n = 176)		
						–	–
Bicarbonate (mmol/L) (n = 1817)	30 (n = 223)	29 (n = 559)	30 (n = 548)	29 (n = 311)	28^a^ (n = 176)		
Base excess (mmol/L) (n = 1810)	6.1 (n = 222)	5.6 (n = 558)	5.8 (n = 545)	5.1 (n = 311)	3.4^a^ (n = 174)	–	–
Rumen chloride (mmol/L) (n = 1761)	36 (n = 220)	38 (n = 565)	40 (n = 524)	38 (n = 282)	35 (n = 170)	–	–

^a^Difference to cows with no comorbidities, P < 0.05, Kruskal Wallis test with Bonferroni correction

^b^Difference to cows with 2 comorbidities, P < 0.05, Kruskal Wallis test with Bonferroni correction

### Diagnoses based on clinical, ultrasonographic, intraoperative and postmortem findings

Based on clinical findings, a definitive diagnosis was made in 1009 (75.0%) of 1341 cows with LDA and in 144 (22.5%) of 641 cows with RDA/AV (P < 0.01, chi^2^ = 286) (Table 10). Clinical differentiation of RDA (n = 338) and AV (303) was not possible. Ultrasonography contributed substantially to the final diagnosis in 22.0% of the cows with LDA and in 53.0% of the cows with RDA/AV, because the clinical findings only allowed a tentative diagnosis. In 3.0% of cows with LDA and in 24.0% of the cows with RDA/AV, a definitive diagnosis was made only via laparotomy, and in 0.5% of the cows with RDA/AV, postmortem examination was necessary to make a diagnosis. Differentiation of RDA and AV was possible only at surgery or postmortem examination.

**Table 10:** Diagnosis after clinical, ultrasonographic and postmortem examination and surgical exploration in 1982 cows with LDA, RDA and AV

Diagnosis based on	LDA (n = 1341) %	RDA/AV (n = 641)^1^	Total (n = 1982)	Chi^2^	Additional tests	P
CE (n = 1153)	1009 (75%)^a^	144 (22.5%)^b^	1,153 (58.1%)	286	Boph	< 0.05
CE + US (n = 1783)	290 (22%)^a^	340 (53%)^b^	630 (31.8%)			
CE + US + L (n = 1979)	42 (3%)^a^	154 (24%)^a^	196 (9.9%)			
CE + US + L + PME (n = 1982)	0 (0%)	3 (0.5%)	3 (0.2%)			

Boph Bonferroni post hoc test, Within rows, values with different superscripts are different (P < 0.05)

*CE* clinical examination, *US* ultrasonographic examination, *L* Laparotomy, *PME* postmortem examination

^1^338 cows with RDA and 303 with AV

## Discussion

In our patient population of cows with displaced abomasum, LDA was four times more common than RDA (67.6 vs. 17.1%) and 4.4 times more common than AV (67.6 vs. 15.3%). This distribution was similar to previously reported ratios of LDA to RDA of 4:1 [40] and 4.7:1 [14]. A ratio of 7.4:1 for LDA to AV was described for North America [40]. The ratio of RDA to AV was 1.1:1, which was similar to 0.9:1 reported by others [11, 12]. Earlier studies reported that 80.2% [41] and 87.5% of cases of LDA occurred in the first 4 weeks postpartum [42], which is comparable to our value of 83.4%. We found that in 82.4% of cows with RDA and 70.5% of cows with AV, the displacement happened in the first 4 weeks postpartum, which differed from 52.6% reported for RDA [14] and 52.5% for AV [41]. Cows with RDA and AV were more often pregnant than cows with LDA (20.9% and 21.2% vs. 7.4%), which was largely in agreement with earlier reports of 11.9% and 7.9%, respectively, for cows with AV and LDA [41] and 18.4% and 9.0%, respectively, for cows with RDA and LDA [14]. The mean duration of illness at the time of referral was significantly shorter in cows with RDA and AV compared with cows with LDA (3.0 and 2.8 days vs. 4.5 days), 36.2% of cows with LDA had been ill for at least five days compared with only 17.1% of cows with RDA and 14.3% of cows with AV. Another study reported that at referral, 43.1% of cows with LDA and 25.7% of cows with RDA had been ill for more than five days [14]. The shorter duration of illness in cows with AV reflects the severity of the condition.

A moderately to severely abnormal demeanour occurred significantly more often in cows with AV (75.9%) than in cows with RDA (58.6%) or LDA (39.4%). Interestingly, other authors observed considerably fewer cows with LDA (7.4%) [14] and RDA (23.5%) [14] with an impaired general condition. The clinical picture of cows with RDA and AV largely depends on the degree of displacement and torsion [7]. Signs of pain were generally rare with bruxism being the most frequent, occurring in 14.7% of cows. Signs of colic were more common in cows with AV (8.3%) and RDA (6.8%) than in cows with LDA (0.7%). Factors involved in visceral pain include overstretching of the abomasal wall, excessive tension on the mesentery and smooth muscle contractions [43]; the first two factors explain the increased incidence of colic in cows with AV. Significantly more cows with AV had moderate to severe tachycardia (27.4%) than cows with RDA (14.2%) or LDA (11.1%); tachycardia is a sign of shock caused by hypovolaemia and intoxication [1]. Compression of the caudal vena cava by the dilated abomasum and sympathetic nerve stimulation also cause an increase in the heart rate [25]. Cows with AV have a greater degree of cardiovascular stress than cows with RDA and LDA and therefore have a higher incidence of enophthalmos (43.9% vs. 32.5 and 30.1%), decreased rectal temperature (31.3% vs. 23.5 and 15.6%), reduced skin surface temperature (57.0% vs. 44.5 and 25.7%), reduced skin turgor (33.6% vs. 25.1 and 25.4%), increased capillary refill time (35.6% vs. 34.5 and 22.5%), foul-smelling breath (27.4% vs. 22.5 and 17.6%) and pale mucous membranes (10.3% vs. 8.1 and 3.5%).

Rumen motility was reduced in 45.5% of all cows and absent in another 44.2%; rumen atony is always a serious clinical sign. Other authors described the rumen motility of cows with LDA as normal or reduced and only rarely as absent [1, 2]. A possible reason for the apparent rumen atony in some of the cows was the displacement of the rumen away from the abdominal wall by the abomasum [9]. Rumen fill as assessed from the left flank was more often reduced in cows with LDA than in cows with RDA or AV (79.7, 71.5 and 61.9%). This was not surprising because cows with LDA had been ill and eating less for a longer period of time than cows with AV. The higher incidence of ruminal tympany in cows with AV (9.9%) and RDA (6.8%) than in cows with LDA (3.0%) can be explained by reflex inhibition of eructation due to pain [44]. BSA and/or PSA were positive in 96.9% of the cows with LDA. These findings are very important for the diagnosis of LDA because they occur in only a few other situations such as rumen atony, displaced gas-filled intestines, pneumoperitoneum and intraperitoneal gaseous abscesses [45]. This supports the statement that a positive BSA and PSA accompanied by audible rumen sounds in the left flank is pathognomonic for LDA [1]. In cows with RDA/AV, BSA and PSA on the right side had an even higher positive result (RDA 98.5, AV 99.3%) than in cows with LDA on the left side (96.9%). However, the diagnostic utility is smaller because several other conditions are associated with these findings including small intestinal ileus, caecal dilatation, diarrhoea, peritonitis and ascites. Differentiation of RDA and AV is not possible based on BSA and PSA.

Similar to rumen atony, intestinal atony is always a serious clinical sign and it was not surprising that this was significantly more common in cows with AV (20.2%) and RDA (15.3%) than in cows with LDA (4.2%). The higher incidence of intestinal atony in cows with AV was why no faeces were found in the rectum of 20.9% of those cows. This was significantly less common in cows with RDA (14.8%) and LDA (5.8%). Palpation of the displaced abomasum transrectally is an important finding, which was almost twice as common in cows with AV than in those with RDA (22.1 vs. 11.2%) and occurred in only 1.1% of cows with LDA. However, these frequencies are considerably lower than those reported by others for RDA (30.8%) [12] and AV (57.5%) [12], (76.3%) [25]. According to one author, the abomasum can always be palpated in cows with RDA and an experienced examiner should be able to identify congested blood vessels in the abomasal wall [7]. The abomasum is large and distended in cows with AV and therefore more readily palpated transrectally. This can also lead to abdominal distension in the right flank and a tense abdominal wall. The right flank was significantly more often distended (17.2 vs. 8.9%) and abdominal guarding was significantly more common (61.1 vs. 47.9%) in cows with AV than in cows with RDA. However, in agreement with other authors [5, 46], reliable clinical differentiation of RDA and AV was not possible in our patients. Ketonuria was significantly more common in cows with LDA (58.3%) than in cows with RDA (33.8%) and AV (23.4%). This was similar to an earlier report of ketonuria in 51.9% of cows with LDA and 19.4% of cows with RDA [14] but it was not in agreement with a reported frequency of 90% in 48 cows with RDA [16]. Left displaced abomasum is common in the early postpartum period and associated with a longer period of reduced feed intake compared with AV, which may explain the higher incidence of ketonuria.

Diagnostic sensitivities > 90% were calculated only for the four variables abnormal demeanour, reduced or absent rumen motility and positive BSA/PSA on the left for LDA and on the right for RDA and AV. From a clinical point of view, only the high sensitivity of positive BSA/PSA is of importance because abnormal demeanour and poor rumen motility are non-specific findings.

The initial effect of abomasal reflux syndrome is an increase in the rumen chloride concentration, which is followed by hypochloraemia, an increase in serum bicarbonate, base excess, hypercapnia, metabolic alkalosis, hypokalaemia, haemoconcentration and azotaemia [30]. More cows with LDA had increased rumen chloride concentration than cows with RDA and AV (78% vs. 51 and 39%). The main reason for this is that LDA has a more protracted course and therefore a larger amount of hydrochloric acid refluxes into the rumen. Furthermore, the reflux may be hindered in cows with AV because of closure of the reticulo-omasal^5^ or omaso-abomasal orifice [30] attributable to the torsion. The three groups differed significantly with regard to acid–base status but the numerical differences were small. 67% of all cows had increased base excess and metabolic alkalosis but only 35% had an increase in blood pH as a result of decompensation. Another study found no difference in mean blood pH between cows with LDA and AV but the base excess was significantly greater in the latter (0.7 vs. 5.3 mmol/L) [47]. Metabolic acidosis with a blood pH < 7.4 occurred in 32% of all cows; this may have been caused by a severe and protracted disease course accompanied by dehydration, anaerobic metabolism and acute septic shock, overriding the metabolic alkalosis as part of a mixed acid–base disturbance [7, 11, 24, 48]. The significantly increased L-lactate concentrations in 78.7% of cows with RDA and 42.6% of cows with AV were due to anaerobic metabolism, which depended on the degree of displacement, the presence of volvulus and the associated hypoxic damage to the abomasal wall [47, 49]. The observation that the L-lactate concentration was higher in the right gastroepiploic vein than in the jugular vein in cows with AV suggests abomasal ischaemia as the cause [47]. An increase in the L-lactate concentration signals a poor prognosis [26, 27] and according to one group, surgery is contraindicated in cows with a lactate concentration ≥ 6 mmol/L [27]. A recent study showed that the L-lactate concentration measured six hours after laparotomy is a reliable prognostic indicator after emergency abdominal surgery in cows [50]. In our experience, the prognosis of a seemingly hopeless situation can be improved when the L-lactate concentration is quickly lowered with aggressive fluid therapy.

Haemoconcentration was significantly more frequent in cows with RDA and AV than in cows with LDA (61.0 and 63.0% vs. 45.0%), which was in agreement with earlier reports of haemoconcentration in cows with RDA [1, 2] and AV [1, 2, 49]. Haemoconcentration is the result of progressive dehydration, which is a typical feature of acute AV, and abomasal sequestration of fluid and reduced water intake in affected cows [25]. Prolonged abomasal reflux in cows with LDA is a major factor in dehydration. Dehydration was also reflected by the serum urea concentration, which was increased significantly more often in cows with AV than in cows with RDA and LDA (61 % vs. 43 and 21%). Other authors reported similar findings [14]. Prerenal azotaemia was caused by progressive dehydration and a decrease in renal perfusion [30].

An increase in the activities of γ-GT (61%), AST (81%), GLDH (84%) and SDH (98%) was seen in the cows with abomasal displacement. Similar findings have been reported previously [17, 51–53] and were interpreted as a result of a negative energy balance, hepatic lipidosis or inflammatory processes. Together with creatine kinase, AST is an important muscle enzyme, and increased activities can result from prolonged recumbency. The most pronounced difference among the three groups of cows was established for the activity of γ-GT, which was elevated in 84, 79 and 52% of cows with AV, RDA and LDA, respectively. The elevated γ-GT activities in cows with AV and RDA were thought to be due to biliary obstruction attributable to duodenal displacement and distortion, and hepatic congestion and hypoperfusion caused by dehydration and hypovolaemia [12]. Hypocalcaemia occured in 78% of all cows. This was in agreement with the results of others studies [54–56], which suggested that subclinical hypocalcaemia plays a role in abomasal displacement.

A definitive diagnosis of LDA could be made in 75% of all cows based on the results of clinical examination alone. In 25% of the cows, the diagnosis was uncertain because the results of BSA and/or PSA were also positive on the right side, or rumen atony was present and the results of double auscultation of the rumen could not be interpreted. An uncertain diagnosis was rarely attributable to an inexperienced clinician because supervision by an expert was standard procedure. When ultrasonographic findings were included, the diagnosis could be made in 97% of cases.

The diagnostic capabilities of ultrasonography using 3.5- and 5.0 MHz linear and convex transducers did not differ from 1998 to 2016. Although the quality of ultrasonograms markedly improved in the study period, the ultrasonographic findings in cows with LDA, RDA and AV did not change. Ultrasonography provides valuable information about the position and size of the abomasum [20–22]. One study stressed the importance of visualisation of the pyloric canal for the diagnosis of LDA [22]. The diagnostic accuracy could be further improved using a combination of abomasocentesis and ruminocentesis and pH measurement of the aspirated fluid [57]. Our study relied on laparotomy in only 40 cows (3%) to confirm the diagnosis of LDA. Reasons for ambiguous ultrasonographic findings included poor image quality because of obesity and accumulation of fluid, gas or fibrin in the abdomen. In contrast to LDA, a definitive clinical diagnosis of RDA and AV could only be made in 22.5% of all cases, which was likely because of other conditions that produce positive BSA and PSA findings on the right side [1, 46, 54]. In contrast, a definitive clinical diagnosis was made in 89.6% of 77 cows with RDA [54] and in 96.3% of 80 cows with AV [25]. Pneumoperitoneum, ileus, gas- or fluid-filled intestines, ascites and caecal dilatation should be considered in the differential diagnosis of RDA/AV [46]. We found that the diagnostic utility of ultrasonography was greater in cows with RDA/AV than in cows with LDA because it almost tripled the diagnostic rate to 75.5%. Similar to an earlier study [23], ultrasonographic differentiation of RDA and AV was not possible, and laparotomy or postmortem examination was necessary to achieve this.

The frequent occurrence of concomitant diseases is in agreement with the findings of other studies, which were recently discussed [59, 60] and will not be expanded on here.

## Conclusions

The occurrence of numerous abnormal clinical and laboratory variables differed significantly in cows with LDA, RDA and AV. A clinical diagnosis based on clinical examination was possible in 75.0% of cows with LDA but in only 22.5% of cows with RDA/AV. Ultrasonography allowed a definitive diagnosis in another 53.0% of the latter, but laparotomy was required in 24.0% and postmortem examination in 0.5%. Laparotomy or postmortem examination is required for the differentiation of RDA and AV. Ultrasonography is useful to confirm a tentative diagnosis of LDA and RDA/AV. Laboratory findings have little diagnostic utility but they allow the assessment of the severity of the illness and in unclear cases may provide information about abomasal reflux syndrome. Differentiation of RDA and AV is not possible based on clinical, ultrasonographic and laboratory findings, and laparotomy or postmortem examination is required.

## Data Availability

The datasets used and analysed for this study are available from the corresponding author on reasonable request.

## Footnotes

1 Diagnostic specificity, positive and negative predictive values and likelihood ratio_+_ could not be calculated because this was a retrospective study without healthy controls.

2 In a cow with a sawhorse stance, the front feet are extended forward and the hind feet are positioned further back than normal, and there is lordosis.

3 When viewed from behind, the abdominal contour is apple-shaped on the left and pear-shaped on the right, giving rise to the term *papple,* derived from pear and apple.
